# Confidential unit exclusion and blood safety

**DOI:** 10.1590/S1516-31802011000500013

**Published:** 2011-09-01

**Authors:** Seyed Moayed Alavian

**Affiliations:** I MD, PhD. Professor of Gastroenterology and Hepatology, Baqiyatallah Research Center for Gastroenterology and Liver Diseases, Tehran, Iran.

Dear Editor,

I read with interest the article by Kasraian et al., recently published in your journal.^1^ They concluded that because of the higher prevalence of HBS, HCV and HIV positivity among blood donors who chose the confidential unit exclusion (CUE) option, offering CUE to blood donors could be a potentially useful method for improving blood safety, since it could increase the detection of infected blood during the window period.

I would like to open the issue for further discussion. Iran is an area of low endemicity for HCV infection, and the most common risk factors are histories of blood transfusion and intravenous addiction.^2,3^ However, for HBV infection, the most common risk factor is transmission during childhood, and most HBV-infected patients are unaware of their infection.^4^ Donor selection is an important strategy for blood safety, but it is not enough! CUE, in which the physician asks the donor whether he or she wants his/her blood to be used for transfusion or not, has been added in order to obtain safer blood and blood products. The donor is asked to choose "*Use my blood"* if he/she has given truthful answers to the questions and, if the questions have not been answered truthfully, is asked to choose "*Do not use my blood"*. Although applying additional policies to maintain safety may lead to more donor rejection and to the exclusion of more blood for transfusion, these policies may vary for each region. Donor screening through physician assessment is still one of the most important components contributing towards providing healthy blood and blood products. The effectiveness of this CUE policy has been unclear. In a study performed by Kean et al. in the United States, 50% of the donors who chose *"Do not use my blood"* had made this choice mistakenly or due to lack of knowledge about this choice.^5^

Finally, I think that the effectiveness of confidential self-exclusion (CSE) systems depends on the risk factors for transfusion-transmitted infections and the educational level of blood donors in each country.
